# Association between combined oral contraceptive pills and depression in women with polyendocrine metabolic ovarian syndrome (formerly polycystic ovary syndrome): a comparative cross-sectional study

**DOI:** 10.1186/s12905-026-04641-6

**Published:** 2026-07-03

**Authors:** Purikorn Prommani, Areepan Sophonsritsuk, Ratana Saipanish, Makaramas Anantaburana, Siriluk Tantanavipas

**Affiliations:** 1 Department of Obstetrics and Gynecology, Faculty of Medicine Ramathibodi Hospital, Mahidol University, Bangkok, Thailand; 2 Reproductive Endocrinology and Infertility Unit, Department of Obstetrics and Gynecology, Faculty of Medicine Ramathibodi Hospital, Mahidol University, 270 Rama VI Road, Rajataewe, Bangkok, 10400 Thailand; 3 Department of Psychiatry, Faculty of Medicine Ramathibodi Hospital, Mahidol University, Bangkok, Thailand

**Keywords:** polyendocrine metabolic ovarian syndrome (PMOS), polycystic ovary syndrome (PCOS), combined oral contraceptive (COC), depression, Patient Health Questionnaire-9 (PHQ-9), testosterone levels, Thai women, mental health

## Abstract

**Background:**

The prevalence of depression among women with polyendocrine metabolic ovarian syndrome (PMOS), formerly polycystic ovary syndrome (PCOS), is high. Combined oral contraceptives (COCs) are widely recommended for the treatment of women with PMOS, potentially reducing stress related to managing PMOS symptoms and improving body image. However, concerns exist regarding a potential association between COC use and depression. Given these conflicting effects, the present study aimed to evaluate the relationship of COCs on depression and its associated factors in Thai women with PMOS, compared with non-COC users.

**Methods:**

A comparative cross-sectional study was conducted among 96 participants (48 COC users and 48 non-COC users) diagnosed with PMOS using the Rotterdam criteria. Depression was assessed using the Thai version of the Patient Health Questionnaire-9 (PHQ-9). Group comparisons of depression prevalence were analysed using the chi-square test. Predictive factors and interaction analyses were performed using multivariable and Firth’s logistic regression, expressed as odds ratios (ORs) and 95% confidence intervals (CIs).

**Results:**

The prevalence of depression was 37.50% of COC users and 35.42% of non-COC users, with no statistically significant association observed (p-value = 0.832). COC use was not associated with depression. Low testosterone levels were the crucial variables associated with depression among women with PMOS (OR 4.38, 95% CI: 1.45–13.28; *p* = 0.009), remaining significant after adjustment for age (OR 5.98, 95% CI: 1.83–19.52; *p* = 0.003). These associations did not affect the interaction of COC use.

**Conclusions:**

Depression is highly prevalent among women with PMOS, with prevalence similar between COC users and non-users. Routine screening is recommended for early detection and timely management, regardless of the treatment approach or COC use. Low testosterone levels may be associated with depression in women with PMOS. However, the findings should be interpreted with caution owing to limited statistical power. Larger prospective studies are required to clarify the association between COC use, androgen imbalance, and depression in women with PMOS.

**Supplementary Information:**

The online version contains supplementary material available at https://doi.org/10.1186/s12905-026-04641-6.

## Background

Polyendocrine metabolic ovarian syndrome (PMOS), formerly polycystic ovary syndrome (PCOS), is a common gynaecological condition in women of reproductive age. The incidence of PMOS, diagnosed according to the Rotterdam criteria, is up to 5–13% of women of reproductive age worldwide [1]. The aetiology of PMOS is multifactorial and not fully understood. Hyperandrogenism (HA), insulin resistance (IR), dysfunction of the hypothalamus-pituitary-ovarian axis, and genetic factors are defined as possible pathogeneses for PMOS. Women with PMOS typically experience irregular menstrual cycles, hyperandrogenic symptoms, and infertility, along with long-term risk, including metabolic and mood disorders [1].

Depression is a common mental disorder affecting all aspects of life and may lead to suicide. Women with PMOS experienced depressive symptoms in approximately 34.8%, varying by studies, ethnicity, and measurement tools [2], which is higher than that observed in the general population. The pathophysiology remains unclear, involving both biological and psychosocial factors such as environmental stress and negative body image. Biological mechanisms in PMOS, including insulin resistance, chronic inflammation, overactive hypothalamic-pituitary-adrenal axis, gut microbiome disruptions, neurotransmitter imbalances, and hormone fluctuations, may contribute to depression in this population [3, 4]. In Thai women, as their ethnicity typically exhibits clear skin and less hair growth, negative body image caused by clinical hyperandrogenism, such as acne and hirsutism, may impose a heightened psychosocial impact.

Combined oral contraceptives (COCs) are associated with an increased risk of depression and antidepressant use [5]. However, COCs remain the primary treatment for PMOS, as they regulate menstrual cycles and alleviate HA symptoms like acne, oily skin, and hirsutism [1]. Along with COCs, lifestyle interventions such as diet, exercise, and weight management are also essential treatment options for PMOS. These changes may decrease stress, potentially decreasing the likelihood of developing depression in women with PMOS.

Given the inconsistent findings on the relationship between COC use and depression among women with PMOS, the primary objective of this study was to compare the prevalence of depression between COC users and non-COC users. The secondary objective was to identify factors associated with depression in this population.

## Methods

### Study design and setting

A comparative cross-sectional study was carried out among women with PMOS attending the Endocrine Clinic, Division of Reproductive Endocrinology & Infertility, Department of Obstetrics and Gynecology, Faculty of Medicine, Ramathibodi Hospital, Mahidol University, between March 2022 and June 2023.

The study received approval from the Ethical Clearance Committee on Human Rights Related to Research Involving Human Subjects, Faculty of Medicine Ramathibodi Hospital (MURA2022/79). The research was conducted in accordance with the Declaration of Helsinki. All participants obtained informed consent in writing.

### Participants

The present study included all eligible women diagnosed with PMOS according to the Rotterdam criteria, aged between 20 and 40 years. Women who had consented and had received any type of COCs for the treatment of PMOS for at least 6 months were enrolled in the COC group, and women who did not use any type of COCs were included in the non-COC group. The assignment to COC or non-COC groups was based on a shared decision-making process between patients and their physicians, reflecting the standard practice protocol in the Gynaecologic Endocrine Clinic at Ramathibodi Hospital. Treatment options were discussed according to patient preferences, clinical symptoms, and safety considerations [6]. Exclusion criteria included a prior diagnosis of depression prior to the diagnosis of PMOS, previous use of antipsychotic drugs, serious medical conditions such as cancer or chronic kidney disease, major surgical procedures unrelated to PMOS, and a history of severe stress within the past three months. Severe stress was exemplified by major life events such as the loss of a significant person, divorce, or loss of employment.

### Sample size

Sample size was determined using a two-sample proportion cross-sectional test, based on an exposure proportion of 0.29 and an unexposed proportion of 0.66 [7], with a significance level of 0.05 and 95% power. After accounting for a 10% data loss, 96 participants were required to compare the prevalence of depression between the two groups: 48 participants in the COC group and 48 participants in the non-COC group.

### Data collection

#### Questionnaire

All participants completed two parts of the self-questionnaire in a private room as follows:

1. Fundamental questionnaire: This section collected information on demographic characteristics, socioeconomic status, including marital status, parity, desired fertility, education, occupation, and monthly income. Monthly income was categorised based on the minimum wage threshold of 285 United States Dollars (USD): below minimum wage (< 285 USD) and above minimum wage (≥ 285 USD). In addition, the gynecologic and medical histories were extracted from the medical records.
2. Depressive assessment: The Thai version of Patient Health Questionnaire-9 (PHQ-9), a self-measurement, was used to assess depressive symptoms based on the Diagnostic and Statistical Manual of Mental Disorders, Fourth Edition (DSM-IV) criteria. The PHQ-9 includes nine items scored from 0 (not at all) to 3 (nearly every day), reflecting symptom frequency over the previous two weeks. The cut-off score ≥ 8 showed a sensitivity of 0.89, specificity of 0.65, positive likelihood ratio of 2.56, negative likelihood ratio of 0.16, and diagnostic odds ratio (OR) 25.46 [8, 9].

#### Anthropometric measurement

A single trained assessor, blinded to COC use and depression status, measured height, body weight, and determined body mass index (BMI = kg/m²). Overweight and obesity were defined as BMI 23.0–24.9 kg/m² and ≥ 25.0 kg/m², respectively, according to Asian-specific cut-offs [10]. Waist circumference (WC) was measured midway between the lower rib margin and the iliac crest. The widest part of the buttocks was measured to determine hip circumference (HC). WC/HC determined the waist-hip ratio (WHR). Abdominal obesity was defined as WC ≥ 80 cm or WHR > 0.85.

#### Clinical features of PMOS

A single trained assessor, blinded to COC use and depression status and overseen by a specialist physician, evaluated signs of HA, irregular menstrual cycles as an indication of ovulatory dysfunction (OD), polycystic ovarian morphology (PCOM) by ultrasonography, and acanthosis nigricans as a marker of IR. All participants were categorised into four phenotypes: phenotype-A (HA + OD+ PCOM), phenotype-B (HA + OD), phenotype-C (HA+PCOM), and phenotype-D (OD+PCOM) [11].

HA was diagnosed based on biochemical and/or clinical HA, such as acne, hirsutism, and/or alopecia. The global acne grading system (GAGS) lesions of acne were employed to assess the severity of acne from six locations of the face, chest, and upper back. The total score ranged from 0 to 44 and was classified as follows: 0 for absent, 1–18 for mild, 19–30 for moderate, 31–38 for severe, and > 38 for very severe acne [12]. The modified Ferriman-Gallway (mFG) score was used for hirsutism evaluation. Hirsutism was defined when the mFG score was ≥ 3. A score of 3–7 characterises mild hirsutism, while a score ≥ 8 indicates moderate to severe hirsutism [13]. Alopecia was evaluated using the Ludwig scale and reported as mild, moderate, and severe [14].

Irregular menstrual cycles were occurring more frequently than every 21 days or less frequently than every 35 days, or fewer than 8 cycles annually [11].

PCOM was identified when there were ≥ 20 follicles per ovary or ≥ 10 ml of ovarian volume in at least one ovary [11].

#### Biomarker-related PMOS

All participants had blood collected at 8 o’clock after fasting and were tested for total testosterone (T), sex hormone binding globulin (SHBG), fasting plasma glucose (FPG), insulin, triglyceride (TG), total cholesterol (TC), high-density lipoprotein cholesterol (HDL-C), and low-density lipoprotein cholesterol (LDL-C).

Prediabetes (PreDM) was identified by FPG 100–125 mg/dL, 2 h PG 140–199 mg/dL, or haemoglobin A1c (HbA1c) 5.7–6.4%. Diabetes mellitus (DM) was indicated by FPG ≥ 126 mg/dL, 2 h PG ≥ 200 mg/dL, or HbA1c ≥ 6.5% [15]. Dyslipidaemia (DLP) was defined as TG ≥ 150 mg/dL, TC ≥ 200 mg/dL, HDL-C < 50 mg/dL, LDL-C ≥ 130 mg/dL, or current use of lipid-lowering medication [16]. T and SHBG were measured employing chemiluminescence assays (Alinity, Abbott, Illinois, U.S.A., and Immulite 2000 xpi, Siemens Healthineers Headquarters, Germany, respectively). The free androgen index (FAI) was calculated as T (ng/mL) x 3.47 × 100 /SHBG (nmol/L). FAI > 6.8% was diagnosed as biochemical HA [17]. The homeostasis model assessment of IR (HOMA-IR) was determined using the formula: insulin (µU/ mL) × glucose (mg/dL)/405. IR was defined as a HOMA-IR level greater than 2.77 [18]. Metabolic syndrome (MetS) was identified by the presence of three or more of the following criteria: WC ≥ 80 cm, TG ≥ 150 mg/dL, HDL-C < 50 mg/dL, blood pressure ≥ 130/85 mmHg, and FPG ≥ 100 mg/dL [19].

#### Statistical analysis

Statistical analysis was performed with Stata Version 17. The distribution of continuous variables was assessed using skewness–kurtosis tests and visual inspection of histograms. For comparisons of continuous variables, T-tests and the Mann-Whitney U test were used for normally distributed variables and non-normally distributed variables, respectively. The chi-square test was applied to compare categorical outcomes, including depression status; Fisher’s exact test was used when expected cell counts were less than 5. Continuous variables with a normal distribution were presented as means ± standard deviations (SD), while non-normally distributed variables were reported as medians with interquartile ranges (IQR). Categorical variables were summarized as frequencies and percentages. Biomarkers were naturally log-transformed to normalise skewed distributions. Depression-related variables in women with PMOS were analysed using univariable and multivariable logistic regression, with results reported as OR with 95% confidence interval (CI). Multivariable logistic regression models were adjusted for age or BMI, based on the event per variable (EPV), to prevent overfitting and preserve model stability. Significant associations in logistic regression were further evaluated using Firth’s logistic regression to assess the potential interactions with COC use on depression. Post-hoc power for the interaction analysis was calculated using a Wald-based approach based on the interaction coefficient and its 95% CI from the logistic regression models. A statistical significance level of p-value < 0.05 was employed to establish statistical significance.

## Results

### Socio-demographic characteristics

A total of 96 participants, consisting of 48 in the COC group and 48 in the non-COC group. None of them had a history of smoking, alcohol use, or psychiatric disorders in their families. All COC users received low-dose combined oral contraceptives containing third- or fourth-generation progestins. The median duration of hormonal use among this group was 12.0 months.

Table 1 presents the sociodemographic characteristics of women with PMOS, showing a mean age of 29.05 years. The majority of participants were single (*n* = 84, 87.50%), nulliparous (*n* = 93, 96.88%), and desired fertility (*n* = 57, 59.38%). Additionally, most participants had a bachelor’s degree (*n* = 77, 80.21%), were employed (*n* = 87, 90.63%), and earned above minimum wage (*n* = 95, 98.96%). No significant difference was shown when comparing those socioeconomic variables between the COC and the non-COC groups. Furthermore, none of the participants reported smoking, alcohol consumption, or a history of psychiatric disorders.

**Table 1:** Sociodemographic characteristics of women with PMOS

Characteristics	Total (*N* = 96)	COC group (*n* = 48)	Non-COC group (*n* = 48)	*p*-value
Age (years)	29.05 ± 4.36	29.00 ± 4.26	29.10 ± 4.49	0.908
Marital status				
Single	84 (87.50)	45 (93.75)	39 (81.25)	0.051
Couple/married	11 (11.46)	2 (4.17)	9 (18.75)	
Divorced	1 (1.04)	1 (2.08)	0 (0.00)	
Parity				
0	93 (96.88)	47 (97.92)	46 (95.83)	1.000
≥ 1	3 (3.13)	1 (2.08)	2 (4.17)	
Desired fertility	57 (59.38)	29 (60.42)	28 (58.33)	0.835
Educational level				
Less than a high school	2 (2.08)	2 (4.17)	0 (0.00)	0.713
High school	6 (6.25)	3 (6.24)	3 (6.25)	
Bachelor’s degree	77 (80.21)	38 (79.17)	39 (81.25)	
Above a Bachelor’s degree	11 (11.46)	5 (10.42)	6 (12.50)	
Occupational status				
Unemployed	9 (9.38)	4 (8.33)	5 (10.42)	1.000
Employed	87 (90.63)	44 (91.67)	43 (89.58)	
Monthly income				
Below minimum wage	1 (1.04)	0 (0.00	1 (2.08)	1.000
Above minimum wage	95 (98.96)	48 (100.0)	47 (97.92)	

Statistical tests: An independent t-test was applied to the normally distributed continuous variable (age). The Chi-square test was used for categorical variables, including marital status, desired fertility, and monthly income. Fisher's exact test was applied to categorical variables with small expected counts, including parity, educational level, and occupational status

Data are presented as the mean ± SD or n (%) as appropriate

*Abbreviation*: *PMOS*, polyendocrine metabolic ovarian syndrome; *COC*, combined oral contraceptive

Baseline clinical and metabolic characteristics between groups categorised by COC use status, independent of depression prevalence, are demonstrated in Tables 2 and 3. This observation is and underscores significant clinical and metabolic disparities that may play a role in varying mental health risks. Table 2 illustrates the anthropometric, clinical features, and disease profile of women with PMOS. Among the 96 participants, the median BW, BMI, and HC were 68.85 kg, 27.28 kg/m^2^, and 102.50 cm, respectively. The mean WC and WHR were 87.51 cm and 0.84. The median duration since PMOS diagnosis was 29.00 months. Overweight and obesity were observed in 5 (5.21%) and 63 participants (65.63%), respectively. PMOS phenotype A was the most prevalent, identified in 55 participants (57.29%). Diagnostic criteria were met by 94 participants (97.92%) for OD, 72 (75.00%) for HA, and 81 (84.38%) for PCOM. Among individuals with clinical HA, acne was the most common manifestation, affecting 90 participants (93.75%), with a mean acne score of 11.81. The majority experienced mild acne, observed in 67 participants (69.79%). However, self-reported perception of acne was less prevalent (*n* = 84, 87.50%) in acknowledging the condition. Hirsutism was reported in 80 participants (83.33%), with 40 (41.67%) experiencing mild hirsutism and 40 (41.67%) experiencing moderate to severe hirsutism. Alopecia was less prevalent, affecting 52 participants (54.17%), with the majority experiencing mild forms (*n* = 49, 51.04%). Acanthosis nigricans was found in 37 participants (38.54%). Reported comorbidities were observed in 18 participants (18.75%) with PreDM, 6 (6.25%) with DM, 31 (32.29%) with DLP, 5 (5.21%) with hypertension (HTN), 21 (21.88%) with MetS, 2 (2.08%) with steatotic liver disease (SLD), and 46 (47.92%) with snoring.

**Table 2:** Anthropometric, clinical features, and disease profile of women with PMOS

Characteristics	Total (*N* = 96)	COC group (*n* = 48)	Non-COC group (*n* = 48)	*p*-value
BW (kg)	68.85 [56.85, 83.53]	66.60 [53.55, 73.35]	77.30 [63.73, 88.00]	0.010
BMI (kg/m^2^)	27.28 [22.10, 32.31]	25.88 [21.34, 29.90]	30.11 [24.91, 34.20]	0.005
Overweight	5 (5.21)	3 (6.25)	2 (4.17)	1.000
Obesity	63 (65.63)	27 (56.25)	36 (75.00)	0.053
WC (cm)	87.51 ± 15.28	82.88 ± 13.16	92.15 ± 15.95	0.003
HC (cm)	102.50 [93.00, 111.50]	100.00 [90.50, 106.50]	108.00 [97.00, 114.00]	0.006
WHR	0.84 ± 0.06	0.83 ± 0.05	0.86 ± 0.06	0.011
Duration since PCOS diagnosis (months)	29.00 [15.00, 45.50]	32.50 [21.00, 47.00]	23.5 [8.00, 39.00]	0.062
PCOS Phenotypes				
A	55 (57.29)	31 (64.58)	24 (50.00)	0.274
B	15 (15.63)	8 (16.67)	7 (14.58)	-
C	2 (2.08)	1 (2.08)	1 (2.08)	-
D	24 (25.00)	8 (16.67)	16 (33.34)	-
OD	94 (97.92)	47 (97.92)	46 (97.92)	1.000
HA	72 (75.00)	40 (83.33)	32 (66.67)	0.059
PCOM	81 (84.38)	40 (83.33)	41 (85.42)	0.779
Clinically evaluated Acne	90 (93.75)	45 (93.75)	45 (93.75)	1.000
Mild	67 (69.79)	35 (72.92)	32 (66.67)	0.737
Moderate	20 (20.83)	8 (16.67)	12 (25.00)	-
Severe	3 (3.13)	2 (4.17)	1 (2.08)	-
Acne score	11.81 ± 8.76	12.17 ± 8.60	11.46 ± 9.00	0.694
Self-perceived acne	84 (87.50)	43 (89.58)	41 (85.42)	0.537
Hirsutism	80 (83.33)	40 (83.33)	40 (83.33)	1.000
Mild	40 (41.67)	18 (37.50)	22 (45.83)	0.067
Moderate to severe	40 (41.67)	22 (45.83)	18 (37.50)	-
mFG score	6.71 ± 3.92	6.72 ± 3.89	6.69 ± 4.00	0.959
Alopecia	52 (54.17)	23 (47.92)	29 (60.42)	0.219
Mild	49 (51.04)	22 (45.83)	27 (56.25)	0.436
Moderate	3 (3.13)	1 (2.08)	2 (4.17)	-
Acanthosis nigricans	37 (38.54)	15 (31.25)	22 (45.83)	0.142
Comorbidities				
PreDM	18 (18.75)	7 (14.58)	11 (22.92)	0.296
DM	6 (6.25)	3 (6.25)	3 (6.25)	1.000
DLP	31 (32.29)	11 (22.92)	20 (41.67)	0.049
HTN	5 (5.21)	2 (4.17)	3 (6.25)	1.000
MetS	21 (21.88)	10 (28.57)	11 (18.03)	0.229
SLD	2 (2.08)	0 (0.00)	2 (4.17)	0.495
Snoring	46 (47.92)	19 (39.58)	27 (56.25)	0.102

Statistical tests: An independent t-test was applied to the normally distributed continuous variable, including WC, WHR, acne score, and mFG score. The Mann-Whitney U test was used for non-normally distributed continuous variables, including BW, BMI, HC, and duration since PCOS diagnosis. The Chi-square test was utilized for categorical variables, including obesity, HA, PCOM, self-perceived acne, hirsutism, hirsutism severity, alopecia, acanthosis nigricans, PreDM, DLP, MetS, and snoring. Fisher's exact test was used for categorical variables with small expected counts, such as overweight, PCOS phenotypes, OD, clinically evaluated Acne, acne severity, DM, HTN, and SLD

Data are presented as the mean ± SD, median [IQR], or n (%) as appropriate

*Abbreviations*: *PMOS*, polyendocrine metabolic ovarian syndrome; *COC*, combined oral contraceptive; *BW*, body weight; *BMI*, body mass index; *WC*, waist circumference; *HC*, hip circumference; *WHR*, waist-hip ratio; *OD*, ovulatory dysfunction; *HA*, hyperandrogenism; *PCOM*, polycystic ovarian morphology; *mFG*, modified Ferriman-Gallwey; *PreDM*, prediabetes; *DM*, diabetes mellitus; *DLP*, dyslipidaemia; *HTN*, hypertension; *MetS*, metabolic syndrome; and *SLD*, steatotic liver disease

**Table 3:** Biomarkers related to PMOS

Biomarkers	Total (*N* = 96)	COC group (*n* = 48)	Non-COC group (*n* = 48)	*p*-value
T (ng/mL)	40.35 [30.60, 56.50]	33.50 [23.85, 52.10]	43.15 [36.15, 60.05]	0.005
SHBG (nmol/L)	49.70 [18.70, 127.50]	127.50 [82.70, 231.50]	18.70 [12.45, 33.95]	< 0.001
FAI (%)	3.10 [0.92, 8.16]	0.92 [0.50, 1.68]	8.16 [4.65, 14.19]	< 0.001
FPG (mg/dL)	88.50 [84.50, 94.50]	87.00 [83.00, 91.00]	90.00 [85.50, 97.50]	0.037
Insulin (µU/mL)	13.15 [6.90, 19.40]	10.15 [6.55, 17.00]	15.65 [7.85, 23.75]	0.081
HOMA-IR	2.87 [1.51, 4.48]	2.35 [1.34, 3.74]	3.47 [1.62, 5.40]	0.046
TG (mg/dL)	103.50 [75.00, 152.50]	110.00 [84.00, 164.50]	96.50 [73.00, 144.50]	0.166
TC (mg/dL)	205.31 ± 29.00	205.27 ± 27.94	205.35 ± 30.32	0.989
HDL-C (mg/dL)	59.21 ± 15.79	65.90 ± 16.08	52.52 ± 12.42	< 0.001
LDL-C (mg/dL)	132.31 ± 33.52	123.19 ± 30.51	141.44 ± 34.22	0.007

Statistical tests: An independent t-test was applied to the normally distributed continuous variable, including TC, HDL-C, and LDL-C. The Mann-Whitney U test was used for non-normally distributed continuous variables, including T, SHBG, FAI, FPG, insulin, HOMA-IR, and TG

Data are presented as the mean ± SD or median [IQR] as appropriate

*Abbreviations*: *PMOS*, polyendocrine metabolic ovarian syndrome; *COC*, combined oral contraceptive; *T*, total testosterone; *SHBG*, sex hormone-binding globulin; *FAI*, free androgen index; *FPG*, fasting plasma glucose; *HOMA-IR*, homeostatic model assessment of insulin resistance; *TG*, triglyceride; *TC*, total cholesterol; *HDL-C*, high-density lipoprotein cholesterol; and *LDL-C*, low-density lipoprotein cholesterol

When comparing the two groups, participants in the COC group had significantly lower BW (66.60 kg vs. 77.30 kg; *p* = 0.010), BMI (25.88 kg/m^2^ vs. 30.11 kg/m^2^; *p* = 0.005), WC (82.88 cm vs. 92.15 cm; *p* = 0.003), HC (100.00 cm vs. 108.00 cm; *p* = 0.006), and WHR (0.83 vs. 0.86; *p* = 0.011). DLP comorbidity was also lower in the COC group (22.92% vs. 41.67%; *p* = 0.049).

The biomarkers related to PMOS are reported in Table 3. Notably, the COC group showed more positive effects on biochemical HA, including a lower T level (33.50 ng/mL vs. 43.15 ng/mL; *p* = 0.005), a higher SHBG level (127.50 nmol/L vs. 18.70 nmol/L, *p* < 0.001), and lower FAI levels (0.92% vs. 8.16%, *p* < 0.001), compared to the non-COC group. In terms of glucose homeostasis markers, showing lower FPG (87.00 mg/dL vs. 90.00 mg/dL; *p* = 0.037) and a lower HOMA-IR (2.35 vs. 3.47; *p* = 0.046). However, no significant difference in insulin levels was observed (10.15 µU/mL vs. 15.65 µU/mL; *p* = 0.081). Regarding lipid profiles, the COC group had a significantly higher HDL-C level (65.90 mg/dL vs. 52.52 mg/dL, *p* < 0.001), and a lower LDL-C level (123.19 mg/dL vs. 141.44 mg/dL, *p* = 0.007) compared to the non-COC group. Levels of TG and TC were not statistically significant.

#### Primary objective: prevalence of depression in women with PMOS

According to the PHQ-9 depression assessment, the median score was 5.00 [IQR: 3.00, 8.50] across all participants, 5.00 [IQR: 2.50, 9.00] in the COC group, and 5.00 [IQR: 3.00, 8.00] in the non-COC group. Overall, 35 participants were diagnosed with depression, indicating a prevalence of 36.50%. However, no statistically significant association between the COC and non-COC groups (37.50% vs. 35.42%; OR 1.09, 95% CI 0.44–2.73; *p* = 0.832) was observed (Table 4).

**Table 4:** Prevalence of depression in women with PMOS using PHQ-9

	PHQ-9 scoremedian [IQR]	*P*-value*	Depression†, *n* (%)	Odds Ratio(95% CI)	*P*-value‡
Total (*n* = 96)	5.00 [3.00, 8.50]	-	35 (36.50)	-	-
Non-COC group (*n* = 48)	5.00 [3.00, 8.00]	Reference	17 (35.42)	Reference	Reference
COC group (*n* = 48)	5.00 [2.50, 9.00]	0.988	18 (37.50)	1.09 (0.44–2.73)	0.832

Statistical tests:

*P-value comparing PHQ-9 scores between groups using the Mann–Whitney U test

†Depression defined as PHQ-9 score ≥ 8

‡P-value comparing the prevalence of depression between groups using the chi-square test

*Abbreviations*: *PMOS*, polyendocrine metabolic ovarian syndrome; *PHQ-9*, Patient Health Questionnaire-9; *IQR*, interquartile range; *CI*, confidence interval; *COC*, combined oral contraceptive

#### Secondary objective: factors associated with depression in women with PMOS

**Fig. 1:**
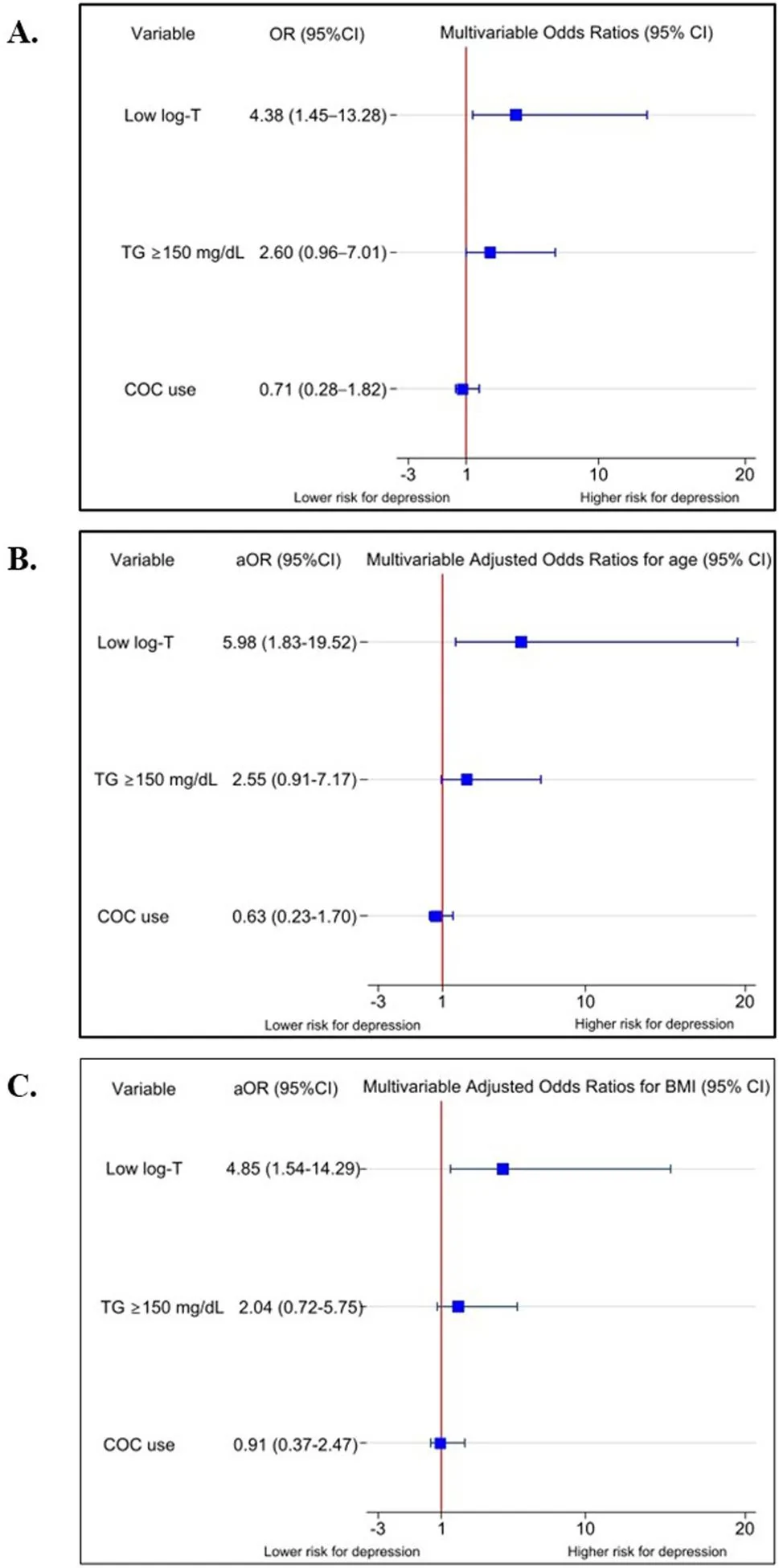
Forest plot of multivariable logistic regression analyses of factors associated with depression in women with PMOS: **A** unadjusted model; **B** age-adjusted model; **C** BMI-adjusted model

All participants were classified into two groups: 35 in the depression group and 61 in the non-depression group. All variables, including COC use and logarithmically transformed biomarkers, were analysed using univariable logistic regression (Supplementary Table S1). Significant variables with *p* < 0.1, including low log-transformed testosterone (log-T), TG ≥ 150 mg/dL, and COC use (the variable of interest), were included in the multivariable logistic regression analysis. Low log-T (OR 4.38, 95% CI: 1.45–13.28; *p* = 0.009) was only identified as an independent factor related to depression among women with PMOS. This association remained after adjusting for age (OR 5.98, 95% CI: 1.83–19.52; *p* = 0.003) and BMI (OR 4.85, 95% CI: 1.54–14.29; *p* = 0.007), as demonstrated in Table 5 and Fig. 1.

**Table 5:** Univariable and multivariable logistic regression analysis of depression in women with PMOS

Variable	Univariable			Multivariable								
				Unadjusted			Model 1*			Model 2**		
	OR	95% CI	*p*-value	OR	95% CI	*p*-value	aOR	95% CI	*p*-value	aOR	95% CI	*p*-value
Low log-T (log_e_ ng/dL)	4.00	1.43–11.11	0.008	4.38	1.45–13.28	0.009	5.98	1.83–19.52	0.003	4.85	1.54–14.29	0.007
TG ≥ 150 mg/dL	2.69	1.04–6.92	0.041	2.60	0.96–7.01	0.059	2.55	0.91–7.17	0.075	2.04	0.72–5.75	0.177
COC use	1.09	0.48–2.51	0.832	0.71	0.28–1.82	0.479	0.63	0.23–1.70	0.363	0.91	0.37–2.47	0.857

Statistical tests: Variables with non-normal distributions (T) were log-transformed to achieve normality. Univariable and multivariable logistic regression analyses were performed, with models unadjusted and adjusted for age and body mass index

*Model 1 was adjusted for age

**Model 2 was adjusted for body mass index

*Abbreviations*: *PMOS*, polyendocrine metabolic ovarian syndrome; *OR*, odds ratio; *aOR*, adjusted odds ratio for age; *CI*, confidence interval; *log-*, log-transformed; *T*, testosterone; *TG*, triglyceride; *COC*, combined oral contraceptive

**Fig. 2:**
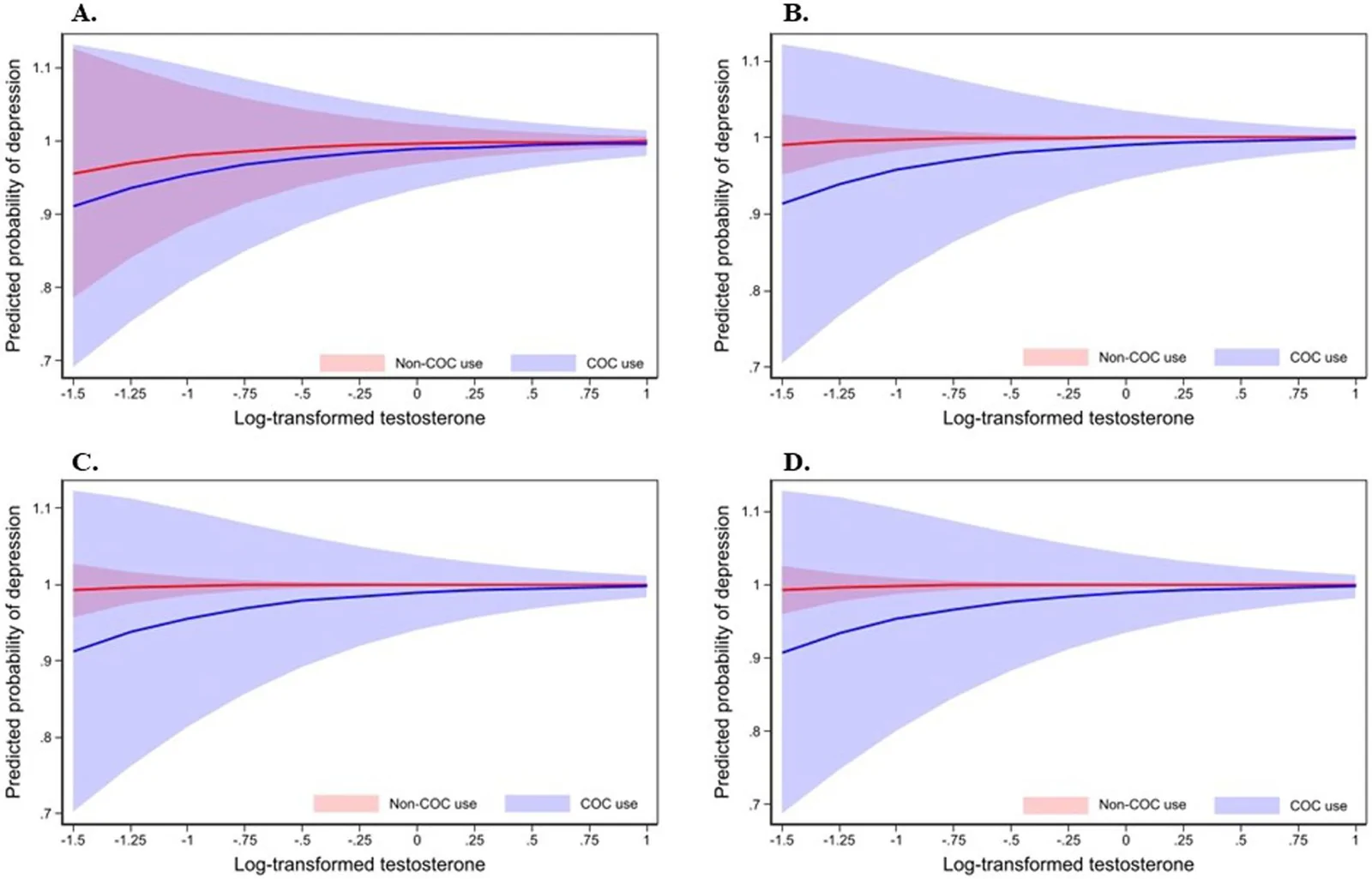
Interaction analysis between log-transformed testosterone levels and COC use on depression in women with PMOS: **A** unadjusted model; **B** age-adjusted model; **C** age and BMI-adjusted model; **D** age, BMI, and desired fertility-adjusted model

Table 6 reports the interaction analysis between low T levels and COC use in relation to depression. Each decrease in log-T was not significantly associated with depression among non-COC users (OR 2.00, 95% CI: 0.91–4.35; *p* = 0.086), but was significantly associated among COC users (OR 1.82, 95% CI: 1.01–3.33; *p* = 0.048). However, the interaction between low T levels and COC use was not statistically significant (OR 1.09, 95% CI: 0.41–2.94; *p* = 0.857). After adjustment for age (Model 1), the associations between low T levels and depression were not observed in either the non-COC or COC use. Further adjustments for age and BMI (Model 2), and for age, BMI, and desired fertility (Model 3), showed that low T levels remained associated with depression in both groups, with no significant interaction between low T levels and COC use. These findings suggest that the association between low T levels and depression did not differ significantly according to COC use. Consequently, the parallel slopes of the predicted depression probability across log-T levels, with overlapping confidence intervals, support the absence of interaction, as shown in Fig. 2. Nevertheless, the study was not primarily powered to detect interaction effects, and post hoc power was approximately 30%. Given the relatively small sample size and limited number of depression events, the interaction analyses should be interpreted with caution, as the absence of statistically significant interactions could be due to limited statistical power and the risk of type II error.

**Table 6:** Interaction analysis between low testosterone levels (log-T) and COC use on depression in women with PMOS

Variable	Unadjusted			Model 1*			Model 2**			Model 3***		
	OR	95% CI	*p*-value	aOR	95% CI	*p*-value	aOR	95% CI	*p*-value	aOR	95% CI	*p*-value
Non-COC use	2.00	0.91–4.35	0.086	2.28	1.18–6.67	0.020	3.03	1.19–7.69	0.019	3.03	1.20–7.69	0.019
COC use	1.82	1.01–3.33	0.048	1.93	1.06–3.45	0.030	1.89	1.03–3.45	0.038	1.85	1.02–3.45	0.042
Interaction	1.09	0.41–2.94	0.857	1.45	0.52–4.06	0.476	1.59	0.54–4.69	0.397	1.62	0.55–4.76	0.385

Statistical tests: Variables with non-normal distributions (T) were log-transformed to achieve normality. Firth’s logistic regression was utilized for interaction analyses. A Wald-based approach was employed for post-hoc power analysis to identify interaction effects, demonstrating approximately 30% post-hoc power

*Model 1 was adjusted for age

**Model 2 was adjusted for age and body mass index

***Model 3 was adjusted for age, body mass index, and desired fertility

*Abbreviation*: *log-*, log-transformed; *T*, testosterone; *COC*, combined oral contraceptive; *PMOS*, polyendocrine metabolic ovarian syndrome; *OR*, odds ratio; *aOR*, adjusted odds ratio; *CI*, confidence interval

## Discussion

The present study found that the COC use exhibited a more favourable overall health profile compared to the non-COC use, including lower BW, BMI, WC, HC, WHR, androgen levels, glucose metabolism, and lipid profile. Acne remained a common characteristic in both groups. Depression was observed in 36.5% of all women with PMOS, which is higher than in the general population. However, no statistically significant association was observed between COC users (37.5%) and non-COC users (35.4%). COC use was not the primary attribute associated with depression in women with PMOS; instead, lower T levels were identified as significant contributors.

Although COC users exhibited a more favourable overall health profile, this finding should be interpreted with caution, given that selection and channeling biases driven by confounding by indication are inherent limitations of the observational study design. In clinical practice, COC prescribing may be influenced by symptom severity and underlying health conditions. Women with more severe hyperandrogenic symptoms, such as severe acne and hirsutism, are more likely to receive COCs, whereas those with metabolic disorders may be less likely. Additionally, obese women may be less likely to use COCs because of cardiovascular risk concerns [6]. Although direct pretreatment symptom severity measures were unavailable, women in the non-COC group had higher BW, BMI, WC, HC, WHR, and a greater prevalence of DLP, indicating systematic baseline differences between groups. This suggests that COC users in our study may represent a healthier subset of the PMOS population. Despite these considerations, the potential benefits of COCs cannot be excluded and should be interpreted with caution. Further studies are required to determine the direct cardiovascular effects of COCs.

In the context of PMOS clinical features, a higher prevalence of acne (93.8%) was observed among women with PMOS in this study compared to a recent meta-analysis by Pourahmad et al. [20], which reported a pooled prevalence of 49%. This may be influenced by ethnicity, the pathophysiology of PMOS, and the performance of diagnostic tools. Although hyperandrogenism is a common feature in PMOS, using GAGS can detect more acne cases than patients report. GAGS’ detailed assessment of lesion types and locations likely explains the higher rate compared to a previous Thai study by Wongwananuruk et al. [21], which reported 77.6% using the facial-focused Leeds system.

The present study confirmed a higher prevalence of depression among women with PMOS compared to the general population, regardless of the method of treatment. It reported a higher rate of depression, at 36.5%, compared to the general Thai women population, which was 2.7–3.4% according to the 2023 Global Burden of Disease study for Thai women aged 15–39 years [22].

A recent meta-analysis study by Infante-Cano M, et al. [2], demonstrated that the mean prevalence of depression among women with PMOS was 34.8%, with variations depending on the self-report questionnaire used. Studies using the PHQ-9 have shown that depression affected between 20% and 64.1%, consistent with the evidence from the present study [23, 24]. However, focusing on Thai women, the present study revealed that Thai women with PMOS frequently reported depression compared to a previous study by Keeratibharat P, et al. [25], which used the Hospital Anxiety and Depression Scale (HADS) and reported a prevalence of only 3.85%. These divergences suggest that not only culture and ethnicity but also the choice of assessment tool may influence the reported burden of depression in women with PMOS. The PHQ-9 assesses a broad range of symptoms that align with the Diagnostic and Statistical Manual of Mental Disorders, Fourth Edition (DSM-IV) criteria for major depressive disorder, whereas the HADS-D focuses on the emotional aspects of depression [26]. Consequently, the PHQ-9 may identify a higher number of patients with depressive symptoms overall.

COCs were not identified as a crucial factor related to or interacting with depression in women with PMOS. A lower rate of major depression has been reported among current COC users compared to former and never users of COCs in American women. However, confounding factors, such as middle income, smokers, and thyroid disorders, may exert a greater influence on mental health than COCs alone [27]. Among women with PMOS, the combination of low-dose COC therapy and lifestyle modifications significantly improved depressive symptoms in overweight or obese individuals by reducing HA and BW [28]. Additionally, COCs may mitigate depression by stabilising hormonal fluctuations from the hypothalamic–pituitary–gonadal axis [29]. These findings may explain why COC use did not have an influence on depression in the present study. Nevertheless, the present study did not identify a correlation between HA, including hirsutism and acne, and depression in women with PMOS, which is inconsistent with the findings of previous studies [30, 31].

Low T levels were a key predictive factor of depression in women with PMOS, independent of COC use. Previous studies have shown that women with depression exhibit lower T levels compared to healthy controls, and testosterone therapy can alleviate depressive symptoms and improve mental health [32, 33]. This effect may be attributed to the anxiolytic and antidepressant properties of testosterone. However, a recent meta-analysis and Mendelian randomisation study by Maharjan DT, et al. [34] exhibited higher T levels in depressed premenopausal women compared with non-depressed women. This finding highlights the impact of testosterone imbalance, abnormally low or high levels, on depression [35]. The complex and bidirectional relationship between T imbalance and depression, along with intriguing biochemical correlates, requires further longitudinal investigation in premenopausal women to understand directionality and underlying mechanisms.

The interaction analysis between T levels and COC use had a post-hoc power of approximately 30%, corresponding to a 70% probability of a Type II error. Therefore, the finding of a non-significant interaction between COC use and T on depression should be interpreted with extreme caution, as we cannot rule out that a true interaction exists.

The strength of this study is that it represents the first comprehensive investigation in Thailand, encompassing a thorough evaluation of all aspects associated with PMOS, including reproductive issues, body image, metabolic factors, comorbidities, and related biomarkers.

A limitation of this study is confounding by indication (channeling bias). Women prescribed COCs were systematically different (healthier, leaner, with different biochemical profiles) from those who were not, making the groups non-comparable. Therefore, the study primarily shows that among women selected for COC use, depression rates are similar to those not selected for it. It cannot robustly assess the causal effect of COCs.

Additionally, the cross-sectional design may introduce selection and recall biases, restricting causal inferences. Although the PHQ-9 is a validated screening tool, it is not a diagnostic tool and may lead to misclassification of depression severity, as well as recall and social desirability biases. Although the sample size was properly calculated, it remains relatively small for a cross-sectional study, potentially underpowering the study to detect modest associations, increasing the risk of a type II error, and limiting subgroup analyses. Therefore, the results should be interpreted with caution. To minimise overfitting and preserve model stability, the primary multivariable model was intentionally parsimonious due to EPV constraints. Consequently, we were unable to adjust for all potentially relevant confounders, including BMI, WC, desired fertility, marital status, and occupational status. Thus, residual confounding by unmeasured or incompletely adjusted factors cannot be fully excluded. Finally, as a single-centre setting at a tertiary care hospital, selection bias and limited generalizability are possible; multicentre studies are recommended to improve external validity. We recommend conducting longitudinal studies to establish the temporal relationships and causality between testosterone levels and depressive symptoms in women with PMOS for future research.

## Conclusion

Women with PMOS exhibited a high prevalence of depression, affecting 36.50% of all participants, 37.50% among COC users, and 35.42% among non-COC users. No statistically significant association was identified between COC use and depression. Given this high burden, universal screening for depressive symptoms in women with PMOS is recommended, regardless of treatment approach, to enable early identification, timely management, and improved mental health outcomes. A potential association between low T levels and depression has been observed. However, these findings should be interpreted cautiously, given the study’s limited statistical power. Large prospective studies are needed to further elucidate the relationship among COC use, androgen imbalance, and depressive symptoms in women with PMOS.

## Supplementary Information

Supplementary Material 1.

## Data Availability

The datasets used and/or analysed during the current study are available from the corresponding author on reasonable request.

## Abbreviations

BMI: Body mass index
CI: Confidence interval
COC: Combined oral contraceptive
DLP: Dyslipidaemia
DM: Diabetes mellitus
DSM-IV: Diagnostic and Statistical Manual of Mental Disorders, Fourth Edition
EPV: Event per variable
FAI: Free androgen index
FPG: Fasting plasma glucose
GAGS: Global acne grading system
HA: Hyperandrogenism
HADS: Hospital Anxiety and Depression Scale
HbA1c: Haemoglobin A1c
HC: Hip circumference
HDL-C: High-density lipoprotein cholesterol
HOMA-IR: Homeostasis model assessment of IR
HTN: Hypertension
IQR: Interquartile range
IR: Insulin resistance
LDL-C: Low-density lipoprotein cholesterol
log-: Log-transformed
MetS: Metabolic syndrome
mFG: Modified Ferriman-Gallway
OD: Ovulatory dysfunction
OR: Odds ratio
PCOM: Polycystic ovarian morphology
PCOS: Polycystic ovary syndrome
PMOS: Polyendocrine metabolic ovarian syndrome
PHQ-9: Patient Health Questionnaire-9
PreDM: Prediabetes
SD: Standard deviation
SHBG: Sex hormone binding globulin
SLD: Steatotic liver disease
T: Total testosterone
TC: Total cholesterol
TG: Triglyceride
USD: United States dollar
WC: Waist circumference
WHR: Waist-hip ratio
