# Supplementary material for: Association between combined oral contraceptive pills and depression in women with polyendocrine metabolic ovarian syndrome (formerly polycystic ovary syndrome): a comparative cross-sectional study
Source: BMC Womens Health. 2026 Jul 3;26:472. doi: 10.1186/s12905-026-04641-6 (PMC13599283; doi:10.1186/s12905-026-04641-6)
Supplement: Supplementary file 1 [file 12905_2026_4641_MOESM1_ESM.docx]

**Supplementary Table S1. Univariable logistic regression analysis for factors associated with depression in women with PMOS**

| **Variable** | **Depression**  (n = 35) | **Non-depression**  (n = 61) | **OR** | **95% CI** | **p-value** |
| --- | --- | --- | --- | --- | --- |
| **Sociodemographic** | | | | | |
| Age group |  |  |  |  |  |
| < 30 years | 28 (44.44) | 35 (55.56) | 1 | - | 0.028 |
| ≥ 30 years | 7 (21.21) | 26 (78.79) | 0.34 | 0.13-0.89 | - |
| Desired fertility |  |  |  |  |  |
| No | 13 (33.33) | 26 (66.67) | 1 | - | 0.599 |
| Yes | 22 (38.60) | 35 (61.40) | 1.26 | 0.54-2.95 | - |
| Employment status |  |  |  |  |  |
| Unemployed | 4 (44.44) | 5 (55.56) | 1 | - | 0.603 |
| Employed | 31 (35.63) | 56 (64.37) | 0.69 | 0.17-2.77 | - |
| Income above minimum wage | | | | | |
| No | 0 (0.00) | 1 (100.00) | - | - | - |
| Yes | 35 (36.84) | 60 (63.16) | Omitted due to perfect prediction | | |
| **Anthropometric measurement** | | | | | |
| Obesity (BMI ≥ 25 kg/m^2^) | | | | | |
| No | 10 (30.30) | 23 (69.70) | 1 | - | 0.366 |
| Yes | 25 (39.68) | 38 (60.32) | 1.51 | 0.62-3.71 | - |
| Abdominal obesity (WC ≥ 80 cm) | | | | | |
| No | 11 (35.48) | 20 (64.52) | 1 | - | 0.891 |
| Yes | 24 (36.92) | 41 (63.08) | 1.06 | 0.47-2.60 | - |
| WHR > 0.85 |  |  |  |  |  |
| No | 19 (33.33) | 38 (66.67) | 1 | - | 0.443 |
| Yes | 16 (41.03) | 23 (58.97) | 1.39 | 0.60-3.23 | - |
| **Clinical features** | | | | | |
| Self-perceived acne |  |  |  |  |  |
| No | 6 (50.00) | 6 (50.00) | 1 | - | 0.303 |
| Yes | 29 (34.52) | 55 (65.48) | 0.53 | 0.16-1.78 | - |
| Clinically evaluated acne |  |  |  |  |  |
| No | 2 (33.33) | 4 (66.67) | 1 | - | 0.870 |
| Yes | 33 (36.67) | 57 (63.33) | 1.16 | 0.21-6.67 | - |
| Hirsutism (mFG score ≥ 3) | | | | | |
| No | 5 (31.25) | 11 (68.75) | 1 | - | 0.636 |
| Yes | 30 (62.50) | 50 (37.50 | 1.32 | 0.42-4.17 | - |
| Alopecia |  |  |  |  |  |
| No | 13 (29.55) | 31 (70.45) | 1 | - | 0.197 |
| Yes | 22 (42.31) | 30 (57.69) | 1.75 | 0.75-4.09 | - |
| Acanthosis nigricans |  |  |  |  |  |
| No | 17 (28.81) | 42 (71.19) | 1 | - | 0.052 |
| Yes | 18 (48.65) | 19 (51.35) | 1.95 | 0.99-5.51 | - |
| PCOS Phenotypes |  |  |  |  |  |
| Non-hyperandrogenic PCOS | 7 (29.17) | 17 (70.83) | 1 | - | 0.393 |
| Hyperandrogenic PCOS | 28 (38.89) | 44 (61.11) | 1.55 | 0.57-4.20 | - |
| Duration since PCOS diagnosis | | | | | |
| < 1 year | 7 (36.84) | 12 (63.16) | 1 | - | 0.969 |
| ≥ 1 year | 28 (36.36) | 49 (63.64) | 0.98 | 0.35-2.77 | - |
| COC use |  |  |  |  |  |
| No | 17 (35.42) | 31 (64.58) | 1 | - | 0.832 |
| yes | 18 (37.50) | 30 (62.50) | 1.09 | 0.48-2.51 | - |
| Duration of COC use |  |  |  |  |  |
| < 1 year | 11 (31.32) | 24 (68.57) | 1 | - | 0.439 |
| ≥ 1 year | 24 (39.34) | 37 (60.66) | 1.42 | 0.59-3.41 | - |
| **Comorbidities** | | | | | |
| preDM |  |  |  |  |  |
| No | 28 (35.90) | 50 (64.10) | 1 | - | 0.812 |
| Yes | 7 (38.89) | 11 (61.11) | 1.36 | 0.40-3.26 | - |
| DM |  |  |  |  |  |
| No | 32 (35.56) | 58 (64.44) | 1 | - | 0.482 |
| Yes | 3 (50.00) | 3 (50.00) | 1.81 | 0.35-9.51 | - |
| DLP |  |  |  |  |  |
| No | 23 (35.38) | 42 (64.62) | 1 | - | 0.752 |
| Yes | 12 (38.71) | 19 (61.29) | 1.15 | 0.47-2.79 | - |
| HTN |  |  |  |  |  |
| No | 33 (36.26) | 58 (63.74) | 1 | - | 0.866 |
| Yes | 2 (40.00) | 3 (60.00) | 1.17 | 0.19-7.37 | - |
| MetS |  |  |  |  |  |
| No | 25 (33.33) | 50 (66.67) | 1 | - | 0.233 |
| Yes | 10 (47.62) | 11 (52.38) | 1.82 | 0.68-4.85 | - |
| SLD |  |  |  |  |  |
| No | 34 (36.17) | 60 (63.83) | 1 | - | 0.691 |
| Yes | 1 (50.00) | 1 (50.00) | 1.76 | 0.11-29.12 | - |
| Snoring |  |  |  |  |  |
| No | 14 (28.00) | 26 (72.00) | 1 | - | 0.075 |
| Yes | 21 (45.65) | 25 (54.35) | 2.16 | 0.93-5.04 | - |
| Any comorbidity |  |  |  |  |  |
| No | 10 (27.78) | 26 (72.22) | 1 | - | 0.174 |
| Yes | 25 (41.67) | 35 (58.33) | 1.85 | 0.76-4.53 | - |
| **Biomarkers** | | | | | |
| FAI |  |  |  |  |  |
| ≤ 6.8% | 22 (33.33) | 44(66.67) | 1 | - | 0.347 |
| > 6.8% | 13 (43.33) | 17 (56.67) | 1.53 | 0.63-3.71 | - |
| FPG |  |  |  |  |  |
| < 100 mg/dL | 29 (35.37) | 53 (64.63) | 1 | - | 0.591 |
| ≥ 100 mg/dL | 6 (42.86) | 8 (57.14) | 1.37 | 0.43-4.33 | - |
| HOMA-IR |  |  |  |  |  |
| ≤ 2.77 | 15 (31.91) | 32 (68.09) | 1 | - | 0.366 |
| > 2.77 | 20 (40.82) | 29 (59.18) | 1.47 | 0.64-3.40 | - |
| TG |  |  |  |  |  |
| < 150 mg/dL | 22 (30.56) | 50 (69.44) | 1 | - | 0.041 |
| ≥ 150 mg/dL | 13 (54.17) | 11 (45.83) | 2.69 | 1.04-6.92 | - |
| HDL-C |  |  |  |  |  |
| ≥ 50 mg/dL | 22 (37.29) | 37 (62.71) | 1 | - | 0.831 |
| < 50 mg/dL | 13 (35.14) | 24 (64.86) | 0.91 | 0.39-2.15 | - |
| LDL-C |  |  |  |  |  |
| < 130 mg/dL | 18 (40.00) | 27 (60.00) | 1 | - | 0.499 |
| ≥ 130 mg/dL | 17 (33.33) | 34 (66.67) | 0.75 | 0.33-1.726 | - |
| Low log-T | - | - | 4.00 | 1.43-11.11 | 0.008 |
| High log-SHBG | - | - | 1.02 | 0.71-1.45 | 0.924 |
| High log-insulin | - | - | 1.48 | 0.85-2.59 | 0.163 |

**Abbreviations:** PMOS, polyendocrine metabolic ovarian syndrome; OR, odds ratio; CI, confidence interval; BMI, body mass index; WC, waist circumference; WHR, waist-hip ratio; mFG, modified Ferriman-Gallwey; COC, combined oral contraceptive; PreDM, prediabetes; DM, diabetes mellitus; DLP, dyslipidaemia; HTN, hypertension; MetS, metabolic syndrome; SLD, steatotic liver disease; FAI, free androgen index; FPG, fasting plasma glucose; HOMA-IR, homeostatic model assessment of insulin resistance; TG, triglyceride; HDL-C, high-density lipoprotein cholesterol; and LDL-C, low-density lipoprotein cholesterol; log-, log-transformed; T, total testosterone; and SHBG, sex hormone-binding globulin.

**Statistical tests:** Variables with non-normal distributions, including T, SHBG, and insulin, were log-transformed to achieve normality. Univariable logistic regression analysis was performed.

**Note:** Data are presented as n (%).
